# Pharmacist follow-up of patients prescribed nirmatrelvir/ritonavir: a single-center retrospective analysis

**DOI:** 10.1186/s40780-025-00507-5

**Published:** 2025-11-19

**Authors:** Ayako Shigeno, Yasukata Ohashi, Ryosuke Masui, Ayako Kiryu, Koji Nagashima, Hirotake Ohashi, Keisuke Seto, Junichi Masuda, Katsuji Teruya, Mugen Ujiie, Norio Ohmagari, Takahiro Nishimura

**Affiliations:** 1 Department of Pharmacy, National Hospital Organization Tokyo Medical Center, 2-5-1 Higashigaoka, Meguro-ku, Tokyo, 152-8902 Japan; 2 Department of Pharmacy, National Center for Global Health and Medicine, Japan Institute for Health Security, 1-21-1 Toyama, Shinjuku-ku, Tokyo, 162-8655 Japan; 3 AIDS Clinical Center, National Center for Global Health and Medicine, Japan Institute for Health Security, 1-21-1 Toyama, Shinjuku-ku, Tokyo, 162-8655 Japan; 4 Disease Control and Prevention Center, National Center for Global Health and Medicine, Japan Institute for Health Security, 1-21-1 Toyama, Shinjuku-ku, Tokyo, 162-8655 Japan; 5 Graduate School of Pharmacy, Tokyo University of Pharmacy and Life Sciences, 1432-1 Horinouchi, Hachioji, Tokyo, 192-0392 Japan

**Keywords:** Nirmatrelvir/Ritonavir, COVID-19, Drug-drug interactions, Cytochrome P450

## Abstract

**Background:**

Nirmatrelvir/ritonavir (NMV/r), a drug used to treat coronavirus disease 2019, has raised concerns about potential drug-drug interactions with concomitant medications due to cytochrome P450 3 A inhibition. However, limited data exist on its use alongside concomitant medications in clinical practice.

**Methods and results:**

In this study, we conducted a retrospective survey using electronic medical records of 32 outpatients whose physicians instructed them to temporarily withdraw some of their regular medications. Medication status and adherence with instructions for the temporary withdrawal of concomitant medications were assessed via telephone. The results showed that three of the 32 patients (9%) took NMV/r contrary to their physician’s instructions, two of whom misused the dosage and one who self-interrupted the treatment. Additionally, 16 patients (50%) did not follow instructions to discontinue their regular medication. Beyond NMV/r adherence, potential post-prescription issues were identified, such as forgetting to resume regular medications or discontinuing medications at the patient’s discretion.

**Conclusion:**

Post-prescription pharmacist follow-ups may improve adherence to NMV/r and support the safe management of regular medications. Future studies are needed to optimize NMV/r use in clinical practice.

## Background

Coronavirus disease 2019 (COVID-19), a global health threat since December 2019, can be effectively managed in high-risk outpatients using nirmatrelvir/ritonavir (NMV/r), an oral antiviral for COVID-19, which reduces the risk of hospitalization or death [1]. The drug received special approval in Japan in February 2022 and is the preferred treatment for patients with mild to moderate disease and risk factors for severe illness, particularly in outpatient settings [2].

Ritonavir, a potent cytochrome P450 (CYP) 3 A inhibitor in NMV/r, boosts nirmatrelvir to maintain effective drug levels against severe acute respiratory syndrome coronavirus 2. However, this inhibitory effect can increase the blood levels of concomitant drugs, leading to toxicity, thereby necessitating temporary discontinuation or dose adjustment to mitigate drug-drug interactions (DDIs) [3, 4]. For many medications, the risk associated with temporary discontinuation is lower than the benefit provided by the antiviral effect of NMV/r. Additionally, because the CYP3A inhibitory effect of ritonavir is diminished by 80% within three days of discontinuation, many drugs can be resumed 3–5 days after NMV/r discontinuation [4, 5]. However, as NMV/r is a specially approved drug, real-world data from 2022 are limited, with no reports from Japan on its use or management alongside regular medications.

The Pharmaceutical Department of the Center Hospital of the National Center for Global Health and Medicine (NCGM) managed DDIs for inpatients and outpatients prescribed NMV/r and conducted telephone follow-ups for outpatients undergoing home treatment [6]. This study focused on outpatients prescribed NMV/r and concomitant DDIs, aiming to clarify the issues associated with NMV/r and the usefulness of pharmacist telephone follow-ups.

## Methods

We retrospectively examined using electronic medical records.

### Patient population and data collection

Outpatients prescribed NMV/r at NCGM from February 15, 2022, to October 14, 2022, who received telephone follow-up to prevent errors related to temporary withdrawal of regular medications affected by DDIs, were included. DDIs between concomitant medications and NMV/r were comprehensively assessed based on information from the search tool we created for pharmacists to check during dispensing [6]. Specifically, this search tool presented the results of evaluations based on the following information sources: the NMV/r Japanese package insert, the Paxlovid™ Fact Sheet for Healthcare Providers [3], the COVID-19 Treatment Guidelines (National Institutes of Health) [7], and the COVID-19 Drug Interactions website (University of Liverpool) [8].Patient background information was examined, including age, sex, COVID-19 risk factors for severe disease [1], body surface area-uncorrected estimated glomerular filtration rate (mL/min), days from symptom onset to NMV/r initiation, number of concomitant medications, and drug classifications. Clinical outcomes included the presence or absence of COVID-19-related hospitalizations or deaths within 28 days of NMV/r initiation.

### Telephone follow-up

Telephone follow-up was conducted for patients deemed to require temporary discontinuation of concomitant medications or monitoring of symptoms due to DDIs with NMV/r [6]. Collected information included duration (in days) and number of telephone calls from the prescription date; adherence to NMV/r and regular medications; adherence to temporary suspension instructions; understanding of when to resume suspended medications; status and number of remaining NMV/r medications; changes in physical condition since initiation; any self-initiated medication changes; and the expected resumption date of suspended medications. A second telephone follow-up was conducted only for patients who showed inadequate understanding during the initial call and those who required follow-up near the expected resumption date of temporarily suspended medications.

### Ethical considerations

This study was approved by the Ethical Review Committee of the NCGM (approval number: NCGM-S-004495-01).

## Results and discussion

Of the 107 outpatients prescribed NMV/r during the study period, 32 (30%) received telephone follow-up; their background characteristics are presented in Table 1. All patients had risk factors for severe disease. Fourteen patients (44%) had moderate renal impairment (BSA-unadjusted eGFR = 30–59 mL/min) and received nirmatrelvir at a reduced dose of 150 mg twice daily. NMV/r was initiated within 5 days of symptom onset for all patients, with a median time from symptom onset to prescription of 1 day (range: 0–3 days), indicating adherence to the dosage and administration precautions in the package insert.

**Table 1:** Patient characteristics

Characteristic	Number of patients prescribed NMV/r (*n* = 32)
Male sex, n (%)	20 (63)
Age, year, median (range)	65 (33-89)
Comorbidity (risk factor), n (%)	
Hypertension	20 (63)
≥60 yr	18 (56)
Diabetes	14 (44)
Obesity (BMI ≥ 25)	9 (28)
Cardiovascular disease	5 (16)
Active cancer	4 (13)
Immunosuppression	3 (9)
History of smoking	3 (9)
Chronic kidney failure	3 (9)
Chronic lung disease	1 (3)
Medical-related technological dependence	1 (3)
eGFR (mL/min), n (%)	
≥ 60	18 (56)
30-59	14 (44)
Days from onset to prescription, median (range)	1 (0-3)
Days from prescription to telephone follow-up, median (range)	3 (1-5)

Abbreviations: BMI: body mass index; eGFR: estimated glomerular filtration rate; NMV/r: nirmatrelvir/ritonavir

The median number of concomitant medications was six (range: 2–14). The most common concomitant medications were angiotensin II receptor blockers (21 patients, 65.6%), calcium channel blockers (20 patients, 62.5%), and statins (19 patients, 59.4%) (Table 2). Five patients (15.6%) were taking medications contraindicated for use with NMV/r, including suvorexant, colchicine, triazolam, estazolam, and clopidogrel. The remaining 27 patients were using medications that require caution when co-administered with NMV/r; these were either temporarily discontinued and resumed a few days after completion of NMV/r, or continued with appropriate dose adjustments and close monitoring of clinical symptoms as necessary. Among the drugs with potential interactions with NMV/r, the most frequently prescribed were amlodipine (16 patients, 50.0%) and rosuvastatin (14 patients, 43.8%) (Table 3).

**Table 2:** Classification of oral concomitant medications (excluding medications used for only one case)

Medication category	Number of patients taking concomitant medications other than NMV/*r* (*n* = 32)
**Cardiovascular drugs**	
ARB	21 (65.6)
Calcium channel blockers	20 (62.5)
Statins	19 (59.4)
β-blockers	4 (12.5)
Diuretics	4 (12.5)
DOAC	3 (9.4)
Omega-3 fatty acids	3 (9.4)
Aspirin	2 (6.3)
Antiarrhythmic drugs	2 (6.3)
**Antigout drugs**	
Xanthine oxidase inhibitors	7 (21.9)
Benzbromarone	2 (6.3)
**Analgesics**	
Acetaminophen	2 (6.3)
Pregabalin	2 (6.3)
**Hypoglycemic drugs**	
Biguanides	7 (21.9)
SGLT-2 inhibitors	5 (15.6)
DPP-4 inhibitors	5 (15.6)
GLP-1 receptor agonists	4 (12.5)
Sulfonylurea	3 (9.4)
Glinide	2 (6.3)
Insulin	2 (6.3)
**Anti-HIV drugs**	
INSTI	4 (12.5)
NRTI	2 (6.3)
**Gastrointestinal drugs**	
PPI/P-CAB	8 (25.0)
Mucoprotectant	4 (12.5)
Clostridium butyricum	3 (9.4)
Laxative	2 (6.3)
**Hypnotic sedatives**,** anxiolytics**	
Benzodiazepine receptor agonists	8 (25.0)
Orexin receptor antagonist	2 (6.3)
**Others**	
Antihistamine	5 (15.6)
LTRA	2 (6.3)
Active vitamin D3 analog	2 (6.3)
Multivitamin ingredients	2 (6.3)

Abbreviations: ARB: angiotensin II receptor blocker; DOAC: direct oral anticoagulant; SGLT-2: sodium-glucose co-transporter II; DPP-4: dipeptidyl peptidase IV; INSTI: integrase strand transfer inhibitor; NRTI: nucleoside reverse transcriptase inhibitor; PPI/P-CAB: proton pump inhibitor/potassium-competitive acid blocker; LTRA: leukotriene receptor antagonist; NMV/r: nirmatrelvir/ritonavir

**Table 3:** Concomitant medications requiring caution due to potential interactions with NMV/r (excluding medications used for only one case)

Medication	Number of patients (*n* = 32)
Amlodipine	16 (50.0)
Rosuvastatin	14 (43.8)
Nifedipine	4 (12.5)
Zolpidem	3 (9.4)
Alprazolam	2 (6.3)
Edoxavan	2 (6.3)
Suvorexant	2 (6.3)

Abbreviations: NMV/r: nirmatrelvir/ritonavir

Medication status and outcomes from telephone follow-up are shown in Table 4. Fifteen patients (46.9%) took NMV/r as directed and understood when to stop and resume their regular medications. Three patients (9.4%) took NMV/r contrary to the physicians’ instructions; two misused the dosage, and one self-discontinued treatment. Sixteen patients (50.0%) did not follow instructions to suspend their regular medications. In brief outpatient consultations with symptomatic patients, it was difficult to fully convey withdrawal instructions. Although this comparison does not directly assess the presence or absence of telephone follow-up, pharmacist telephone follow-up may have contributed to preventing adverse events and improving adherence by avoiding non-compliance with or misinterpretation of physicians’ instructions. A second follow-up call was made to six patients who had not fully understood the initial instructions and needed additional support near the expected resumption date. Of these, five resumed their medications as directed, but one patient remained withdrawn past the scheduled resumption date until prompted by the pharmacist’s follow-up call.

**Table 4:** Medication adherence of patients who received telephone follow-up (*n* = 32)

No. of cases (%)		Correct recognition of temporary discontinuation and resumption of regular medication	
		Yes	No
Correct use of NMV/r	Yes	15 (46.9)	14 (43.8)
	No	1 (3.1)	2 (6.3)

Abbreviations: NMV/r: nirmatrelvir/ritonavir

Table 5 shows six representative intervention cases requiring pharmacist advice from among 32 patients who received telephone follow-up. Two patients (Nos. 2 and 5) withdrew a drug different from that directed by the physician, which the pharmacist corrected. Five patients (Nos. 1, 2, 3, 5, and 6) required clarification on when to resume temporarily withdrawn medication. One patient (No. 6) was instructed to resume the medication immediately, as this was the second follow-up call, and the scheduled resumption date had already passed. Two patients (Nos. 1 and 3) had mistakenly received NMV/r; however, both realized the error and confirmed correct usage upon pharmacist contact.

**Table 5:** Pharmacist intervention with patients whose medication needed to be temporarily discontinued

No.	Age	Number of medications taken daily	Unit-dose packaging of daily medications	Adherence of NMV/*r*	Adherence to daily medications	Understanding of physician’s instructions	Medication adjustment instructions	Problems the patient was having	Details of pharmacist’s telephone follow-up with patients
1	40s	9	No	No	Yes	No	Nifedipine: discontinue Rosuvastatin, Rosuvastatin/Ezetimibe: discontinue from the start of NMV/r until 3 days after the end of the dose	The patient was taking only one nirmatrelvir tablet when he was supposed to take two until the second dose; therefore, we instructed him again on how to take it correctly. Three medications were temporarily discontinued, one of which (nifedipine) the patient did not understand when to resume.	We encouraged him to take his blood pressure at home and explained the planned resumption date. A second phone call was made the day after the scheduled resumption date, confirming that all regular medications had been resumed and that blood pressure was not significantly different from normal.
2	40s	12	No	Yes	No	No	Rosuvastatin: discontinue Budesonide (inhaler); use together with caution	Because the physician explained only verbally to the patient, the patient lost track of the withdrawal instructions after returning home. He remembered that drugs with “ro” were to be discontinued and had withdrawn rosuvastatin and losartan. Budesonide had been discontinued at his own discretion.	We recommended a withdrawal of rosuvastatin only and explained the planned resumption date. A second telephone call was made two days after the scheduled restart date to confirm that the patient was resuming rosuvastatin. We confirmed that budesonide was resumed and that there was no change in asthma symptoms.
3	40s	8	No	No	Yes	No	Triazolam: discontinue	Two ritonavir tablets were mistakenly taken during the first oral dose. Triazolam was withdrawn but was to be resumed the day after NMV/r was completed.	After the second dose, it was confirmed that the correct combination of NMV/r was being taken. Triazolam was resumed 3 days after the end of NMV/r in consideration of the effects of ritonavir, and the patient was instructed to be careful about somnolence when resuming the drug.
4	50s	5	No	Yes	Yes	ー	Suvorexant: discontinue	He was about to stop taking NMV/r of his own accord because his sore throat was not improving.	Since no side effects appeared, we instructed the patient to finish the dose. We explained that he could take the Loxoprofen he had for the sore throat. We confirmed that he was not taking Suvorexant.
5	50s	6	No	Yes	No	No	Candesartan/Amlodipine: No details of instructions are provided	The patient had been told by his doctor to discontinue his antihypertensive medication. He mistakenly thought that topiroxostat was an antihypertensive drug and discontinued it at his own discretion.	We explained that topiroxostat would not affect him and that he should continue taking it. We instructed him to resume the antihypertensive medication while measuring his blood pressure at home.
6	70s	5	No	Yes	Yes	No	Rosuvastatin: discontinue from the start of NMV/r until 3 days after the end of the dose	During the first phone call, the patient was aware of the scheduled resume date. However, a second phone call was made the day after the scheduled resume date, but the patient had not resumed his medication.	During the second phone call, we explained that the scheduled resume date had passed, and the medication was resumed the same day.

Abbreviations: NMV/r: nirmatrelvir/ritonavir

A study by a pharmacist in Malaysia followed up 415 patients on days 3 and 5 after NMV/r prescription, either in person, by video call, or by telephone, to assess medication adherence and monitor side effects [9]. Poor adherence was observed in 13 patients (3.1%) due to intolerable side effects (diarrhea and dysgeusia), medication errors, and self-discontinuation due to fear of side effects or symptom improvement. There were no discontinuations owing to side effects; however, similar trends were observed for medication errors and self-discontinuation. Regarding regular medications, a previous study [9] reported the results of a survey on compliance with temporary withdrawal instructions based on the University of Liverpool’s COVID-19 drug interaction database [8] but did not provide information on whether patients successfully resumed their regular medications as directed by physicians. In Japan, Mutoh et al. reported conducting telephone calls to assess outcomes during the first 28 days after prescription [10]; however, details of instructions regarding compliance with NMV/r medications and temporary suspension of regular medications were not provided. Although following up with all patients prescribed NMV/r via telephone proved challenging, this study focused on DDIs with regular medications, which require more intervention by healthcare professionals, and followed through to the resumption of temporarily discontinued medications after completing NMV/r treatment. This study revealed adherence patterns to NMV/r and potential post-prescription issues, including forgetting to restart regular medications and patient-initiated discontinuation of medications that should have been continued. One possible reason for forgetting to restart regular medications is the required 3-day interval between NMV/r completion and the resumption of regular medications due to ritonavir’s CYP3A-inhibitory effects [4]. Post-prescription pharmacist follow-up may be beneficial for improving adherence to NMV/r treatment and ensuring proper management of regular medications.

No serious adverse events were observed within 28 days post-prescription among the 32 patients at risk of DDIs with NMV/r. In a previous report, pharmacists’ telephone contacts on days 3 and 5 after discharge, in addition to discharge instructions, significantly reduced the incidence of preventable drug-related adverse events 30 days after discharge [11], supporting the appropriateness of the timing of pharmacist follow-up in this study. Stoiber et al. reported that 80 of 140 patients (57.1%) prescribed NMV/r required pharmacist intervention for DDIs, and 24 (17%) had significant DDIs [12]. DDIs reportedly accounted for 5.7% of cases where physicians refrained from prescribing NMV/r to patients despite clinical need [13]. Pharmacists can play a key role in promoting the appropriate use of NMV/r by screening for DDIs at the time of prescription and providing post-prescription support until safe resumption of regular medications.

The study’s limitations include the small sample size of 32 patients from a single institution, conducted over 8 months immediately following NMV/r approval. Second, post-prescription telephone support was limited to patients who had temporarily discontinued their medication; we could not verify the outcomes of those not contacted or those who may not have adhered to NMV/r despite follow-up. Additionally, adherence to NMV/r after follow-ups could not be fully confirmed. Despite follow-ups, repeated interventions occurred in only six cases, and actual resumption of suspended medication was not verified in all patients. The post-marketing surveillance from February 14, 2022, to August 13, 2022 revealed 716 cases of adverse drug reactions among approximately 29,004 patients [14]. Of these, 68 were considered serious, with hospitalization not entirely excluded, which was also considered a limitation.

In this initiative, telephone calls were the method of post-prescription follow-up. However, in recent years, information and communication technologies, such as electronic medication registries and social networking platforms, have gained prominence. The use of electronic media to collect patient-reported outcomes has shown promise [15]. Incorporating such tools may help reduce the burden on both healthcare professionals and patients, ensuring accurate and efficient follow-up.

## Conclusions

Pharmacist-led follow-up of patients prescribed NMV/r helped identify adherence issues and supported proactive identification and management of DDIs. This collaborative approach supports safer and more effective outpatient COVID-19 care. Future studies should expand on these findings to further optimize the use of NMV/r.

## Data Availability

The datasets used and/or analyzed during the current study are available from the corresponding author on reasonable request.

## Abbreviations

COVID-19: Coronavirus Disease 2019
CYP: Cytochrome P450
DDIs: Drug-Drug Interactions
NCGM: National Center for Global Health and Medicine
NMV/r: Nirmatrelvir/Ritonavir
